# An Efficient and Versatile System for Visualization and Genetic Modification of Dopaminergic Neurons in Transgenic Mice

**DOI:** 10.1371/journal.pone.0136203

**Published:** 2015-08-20

**Authors:** Karsten Tillack, Helia Aboutalebi, Edgar R. Kramer

**Affiliations:** Development and Maintenance of the Nervous System, Center for Molecular Neurobiology, University Medical Center Hamburg-Eppendorf, Hamburg, Germany; University of Chicago, UNITED STATES

## Abstract

**Background & Aims:**

The brain dopaminergic (DA) system is involved in fine tuning many behaviors and several human diseases are associated with pathological alterations of the DA system such as Parkinson’s disease (PD) and drug addiction. Because of its complex network integration, detailed analyses of physiological and pathophysiological conditions are only possible in a whole organism with a sophisticated tool box for visualization and functional modification.

**Methods & Results:**

Here, we have generated transgenic mice expressing the tetracycline-regulated transactivator (tTA) or the reverse tetracycline-regulated transactivator (rtTA) under control of the tyrosine hydroxylase (TH) promoter, TH-tTA (tet-OFF) and TH-rtTA (tet-ON) mice, to visualize and genetically modify DA neurons. We show their tight regulation and efficient use to overexpress proteins under the control of tet-responsive elements or to delete genes of interest with tet-responsive Cre. In combination with mice encoding tet-responsive luciferase, we visualized the DA system in living mice progressively over time.

**Conclusion:**

These experiments establish TH-tTA and TH-rtTA mice as a powerful tool to generate and monitor mouse models for DA system diseases.

## Introduction

The DA system in the brain is essential for mental and physical health as it controls many basic processes including movement, memory, motivation and emotion. Alterations in the DA system can lead to diseases such as Parkinson’s disease, schizophrenia, attention-deficit hyperactivity disorder and drug addiction [1]. The ability to temporarily regulate gene expression in DA neurons would greatly advance the field since it would allow the separation of developmental and adult gene functions, the induction of genetically neurodegenerative, neuroprotective or regenerative processes, and the labelling and monitoring of DA neurons over time during development and aging.

In the last decade several Cre expressing mice have been generated to delete genes of interest in DA neurons using promoters of different genes. They include genes such as TH [2–4], the first and rate-limiting enzyme for dopamine synthesis and highly expressed in DA neurons from early embryonic day 9–10 onwards throughout life [5], dopamine transporter (DAT) [6–10], required for dopamine re-uptake into DA neurons and expressed from embryonic day 9, and Pitx3 [11], a transcription factor involved in DA neurons differentiation. Moreover, even mice with estrogen-regulated Cre under the control of the DAT [12], Pitx-3 [13], or TH promoter [14] were generated for temporarily controlled gene deletions. In these mice, Cre recombinase is fused to a truncated estrogen receptor (CreER) and Cre activation can be regulated with the estrogen receptor antagonist, tamoxifen, which enables translocation of CreER into the nucleus where it can trigger recombination [15]. However, the use of the CreER system is frequently hampered by leakiness, female infertility during tamoxifen treatment, and unexpected toxicity of tamoxifen in the presence of the CreER construct [15–18]. In addition, Cre-mediated recombination is an irreversible event, allowing one to switch genes of interest on or off only once, which prevents switching back and forth between the on and off state.

To overcome this limitation, we established transgenic mice using the tet-system [19, 20] that expresses the tetracycline-dependent transactivator (tTA) or the reverse tetracycline-dependent transactivator (rtTA) under control of mouse TH gene promoter elements. In the tet-OFF system, tTA binds and activates the associated tetO promoter in the absence of tetracycline or its derivative doxycycline (DOX). In the tet-ON mice, rtTA binds and activates the tetO promoter in the presence of DOX. Here, we show that in TH-tTA and TH-rtTA mice, tTA and rtTA are expressed in the midbrain DA system and their activity is tightly regulated by DOX. We used these mice to genetically delete DA neurons and to detect and continuously monitor changes of the DA system over time in living mice. These experiments establish TH-tTA and TH-rtTA mice as an excellent tool for genetically modulating and visualizing the DA system in mice.

## Materials and Methods

### Transgenic mice

All procedures were performed in accordance with the German and European Animal Welfare Act and approved by the Hamburg State Authority for Health and Consumer Protection (BGV Hamburg, license 31/11; 28/12). All surgery was performed under anesthesia, and all efforts were made to minimize suffering. To obtain tissue for histology, mice were anesthetized by intraperitoneal injection of Ketamine (240mg/kg) and Xylazine (30mg/kg) and subsequently transcardially perfused with buffer and fixative. For stereotactic injection and *in vivo* imaging, isoflurane anesthesia was used in combination with buprenorphine (0,05mg/kg) and carprofen (5mg/kg) analgesia for the stereotactic injection. Surgical anesthesia was ensured by the absence of any nociceptive responses to tail and toe-pinching. Mice not needed for histology were sacrificed by cervical dislocation.

Mice were housed under constant conditions at 22°C and 40–50% humidity in a 12 h light/dark cycle with free access to food and water. Doxycycline (DOX) was administered to mice with the drinking water (2 mg ml-1) plus 5% sucrose or food pellets (200 mg kg-1).

**Fig 1:**
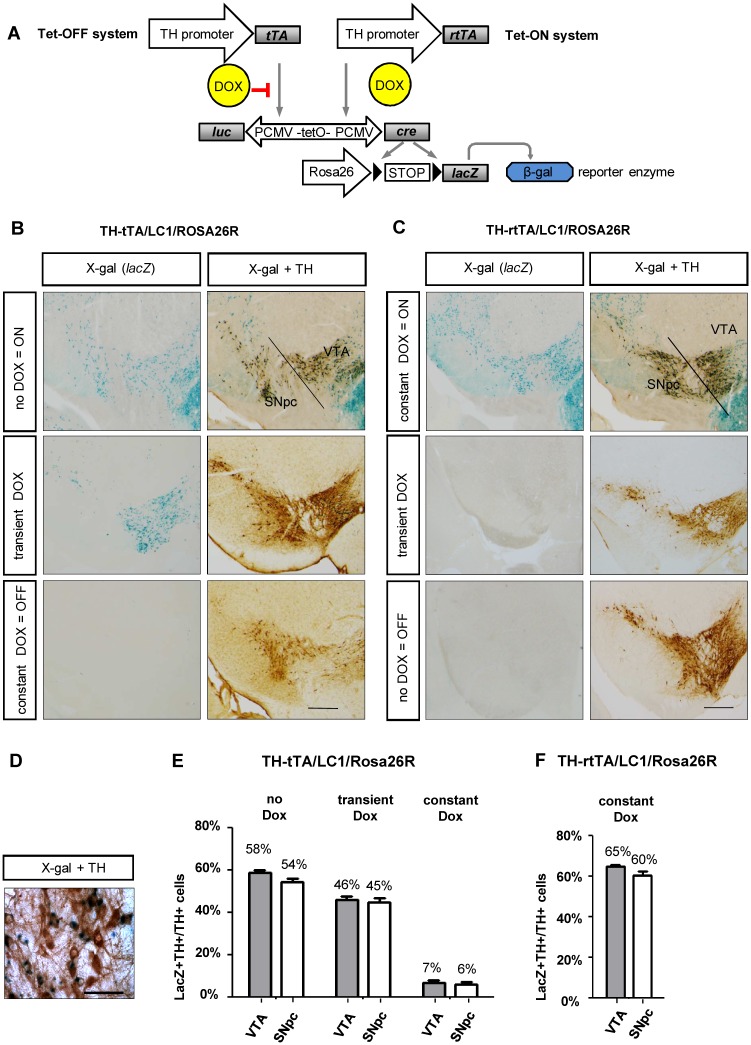
Specificity, inducibility and efficacy of gene expression in TH-tTA/LC1/Rosa26R and TH-rtTA/LC1/Rosa26R mice. **(A)** Genetic scheme of DOX-regulated reporter gene expression in TH-tTA and TH-rtTA mice crossed with LC1 and Rosa26R reporter mice. **(B and C)** Coronal midbrain brain sections with substantia nigra pars compacta (SNpc) and ventral tegmental area (VTA) DA neurons of TH-tTA/LC1/Rosa26R (B) and TH-rtTA/LC1/Rosa26R (C) mice raised with or without doxycycline (DOX) or transiently with DOX to have the system switched off during development (TH-tTA mice treated with DOX during pre- and postnatal development till 6 weeks of age; TH-rtTA mice raised without DOX) and on during analysis (TH-tTA mice from the age of 6 weeks without DOX; TH-rtTA mice from the age of 6 weeks with DOX). Sections were stained for β-galactosidase activity with X-gal to visualize cells with activated tet-system. Adjacent sections were X-gal stained and co-stained with tyrosine hydroxylase (TH) antibodies and DAB substrate (brown) to mark DA neurons. **(D)** Zoom in picture of TH and lacZ double positive cells in the SNpc. **(E and F)** Quantification of TH and lacZ double positive cells (LacZ+TH+) in the SNpc and VTA to estimate recombination efficacy in DA neurons (TH+) of animals treated constantly (constant DOX), transiently (transient DOX) or without DOX (no DOX) in the tet-OFF (E) and tet-ON mice (F). mean + s.e.m.; n = 3. Scale bars: 500 μm (B and C), 50 μm (D).

To generate the TH-tTA mice, the tTA2S encoding sequence from pUHT61-1 was cloned by PCR downstream of a 8.9 kilobase mouse TH promoter fragment in pBS-SK(-)-TH-Cre (W. Wurst, Helmholtz Center Munich, Germany) digested with SmaI and XbaI to linearize the vector and to release the Cre coding sequence (Fig 1, S1 Fig). To generate the TH-rtTA mice, rtTA3G was PCR cloned from pUC57 (GenScript USA Inc.) with primers encoding restriction sites for XmaI at the ends and cloned into XmaI digested pBS-TH-tTA2S to release tTA2S. TH-tTA2S and TH-rtTA3G expression cassettes were released from the pBS vector backbone by SalI digestion and purified by agarose DNA gel electrophoresis for pronucleus injection into C57BL/6JxCBA F1 hybrid zygotes. Positive founders were identified by PCR genotyping using the following primers: gTH2f- AGAACTCGGGACCACCAGCTTG, gTH2r- CACTTTAGCCCCGTCGCGATG and backcrossed to C57BL6 mice. Depending on the founder, the TH-tTA and TH-rtTA mice can be officially called C57BL/6-Tg(TH-tTA2S)1 to 4 and C57BL/6-Tg(TH-rtTA3G)1 to 5, respectively.

**Fig 2:**
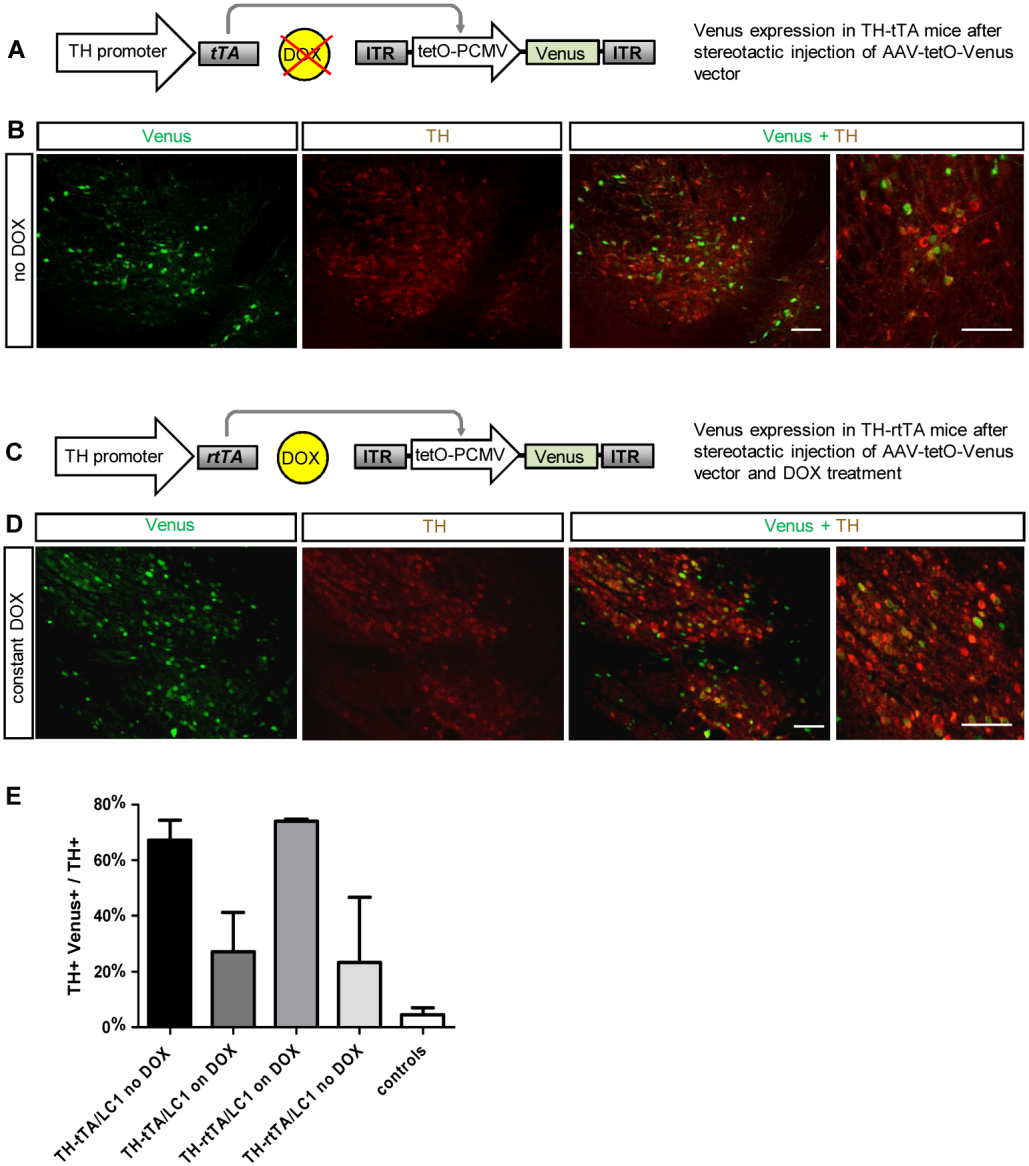
Cre independent adult gene expression in DA neurons of TH-tTA and TH-rtTA mice using a AAV-tetO-Venus vector. **(A)** Scheme of fluorescent DA neuron labelling in non-DOX treated TH-tTA mice stereotactically injected with AAV-tetO-Venus vector in the ventral midbrain. **(B)** Confocal fluorescent pictures of Venus expression (green) in DA neurons co-stained for tyrosine hydroxylase (TH; red) in the substantia nigra pars compacta (SNpc) of sagittal TH-tTA mouse brain sections. **(C)** Scheme of fluorescent DA neuron labeling in DOX-treated TH-rtTA mice stereotactically injected with AAV-tetO-Venus vector in the ventral midbrain. **(C)** Scheme of fluorescent DA neuron labelling in DOX-treated TH-rtTA mice stereotactically injected with AAV-tetO-Venus vector in the ventral midbrain. **(D)** Confocal fluorescent pictures of Venus expression in DA neurons co-stained for TH in the substantia nigra pars compacta (SNpc) of sagittal TH-rtTA mouse brain sections. **(E)** Quantification reveals that in the ON-state 67% and 73% and in the OFF-state 27% and 23% of TH+ cells are also Venus-positive in TH-tTA and TH-rtTA mice, respectively. In non-transgenic mice (controls) only 5% of TH+ cells were also Venus-positive. n = 3–5. Scale bars: 250 μm and in high magnification picture 50 μm.

**Fig 3:**
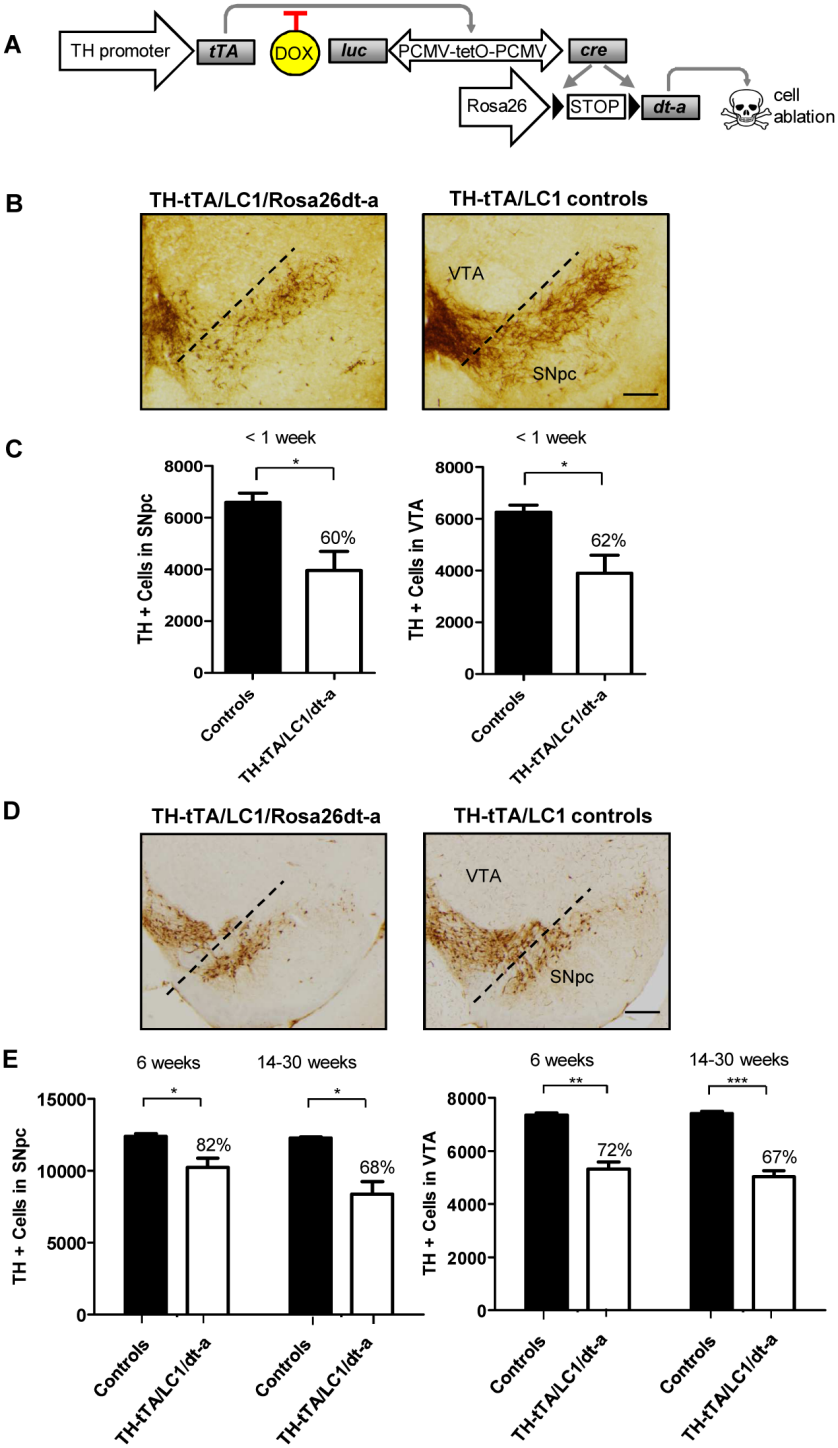
Loss of DA neurons in newborn and adult TH-tTA/LC1/Rosa26dt-a mice. **(A)** Scheme of diphteria toxin-a (dt-a) expression in TH-tTA/LC1/Rosa26dt-a mice. **(B)** Coronal midbrain sections of newborn TH-tTA/LC1/Rosa26dt-a mice and control mice, both stained for tyrosine hydroxylase (TH) to visualize DA neurons of the substantia nigra pars compacta (SNpc) and ventral tegmental area (VTA). No DOX was applied. Scale bar = 50 μm. **(C)** Stereological quantification of TH-positive cells in the SNpc and VTA of newborn TH-tTA/LC1/Rosa26dt-a and control mice without DOX treatment. **(D)** TH stained coronal midbrain sections of adult TH-tTA/LC1/Rosa26dt-a mice and control mice raised with DOX until 6 weeks of age followed by 30 weeks without DOX. Scale bar = 500 μm. **(E)** Stereological quantification of TH-positive cells in the SNpc and VTA of adult TH-tTA/LC1/Rosa26dt-a and control mice raised with DOX until 6 weeks of age followed by 6 or 14–30 weeks without DOX. mean + SEM, *p < 0.05, **p < 0.01, ***p < 0.001 Student´s *t*-test, n = 3.

**Fig 4:**
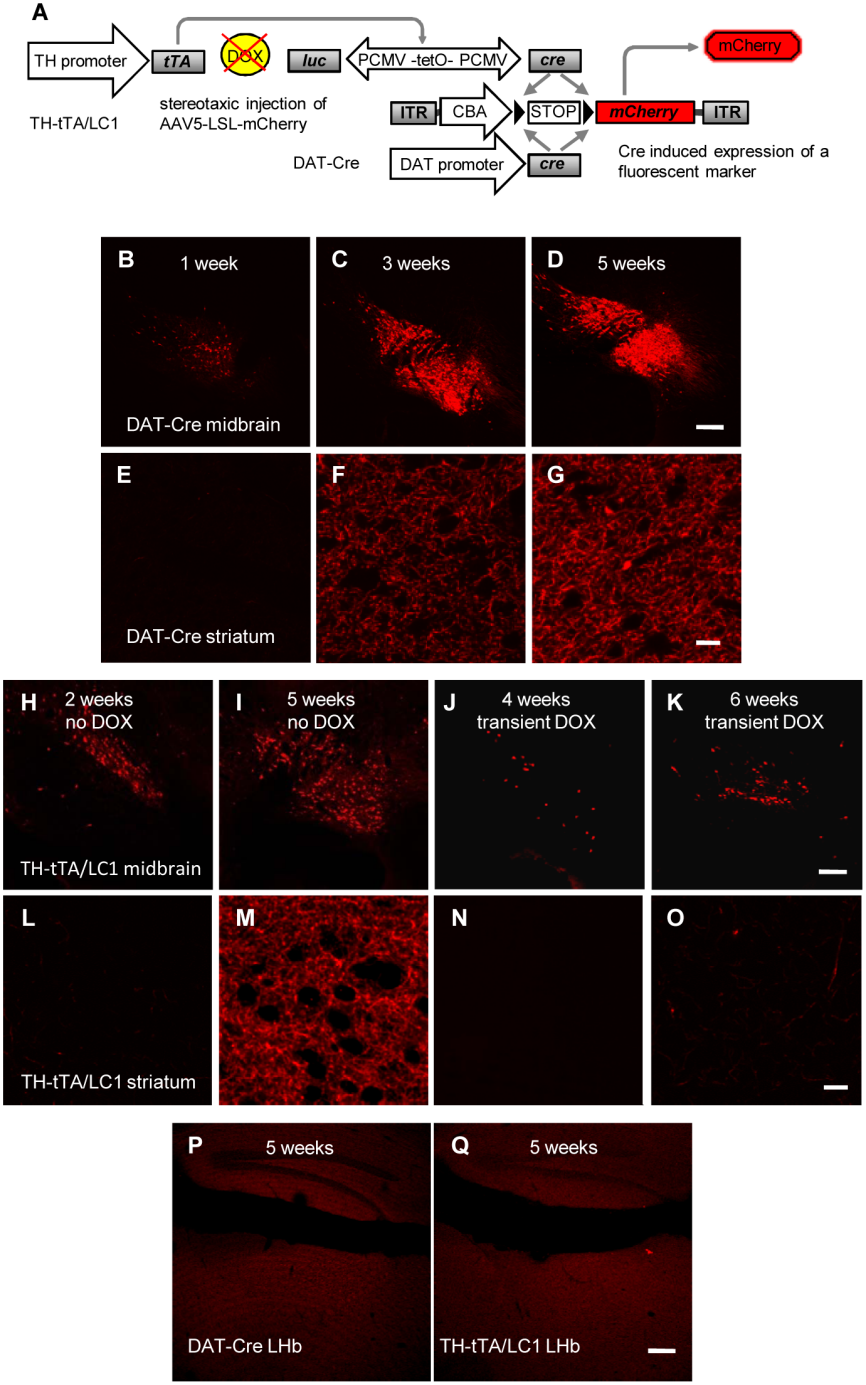
Efficient fluorescent labeling of DA neurons and their axons in AAV-LSL-mCherry injected DA neuron-specific Cre mice. **(A)** Scheme for specific fluorescent labeling of DA neuron in mice expressing a DA neuron-specific Cre and stereotaxic injected in the mindbrain with a recombinant AAV vector expressing mCherry after a floxed STOP codon. **(B-G)** Fluorescent mCherry expression in DA cells of the midbrain (B-D) and DA fibers in the striatum (E-G) in sagittal sections of stereotaxic injected DAT-Cre mice. Expression was evaluated 1 (B,E), 3 (C,F) and 5 weeks (D,G) after injection. After 1 week, no expression could be detected in DA fibers of the striatum. Expression in DA fibers was saturated 5 weeks after injection. Scale bars = 200 μm (B-D); 20 μm (E-G). **(H-O)** Fluorescent mCherry expression in DA cells of the midbrain (H-K) and DA fibers in the striatum (L-O) after stereotaxic injection of TH-tTA/LC1 mice. Fluorescent expression was evaluated at the indicated time points in mice raised without DOX. Some mice were kept on DOX-containing food after the virus injection until analyzed (J, K, N, O). Scale bars = 200 μm (H-K); 20 μm (L-O). **(P and Q)** No fluorescent mCherry expression was detected in the lateral habenula (LHb) of AAV-LSL-mCherry injected DAT-Cre (P) and TH-tTA/LC1 (Q) mice after 5 weeks. Scale bars = 200 μm.

**Fig 5:**
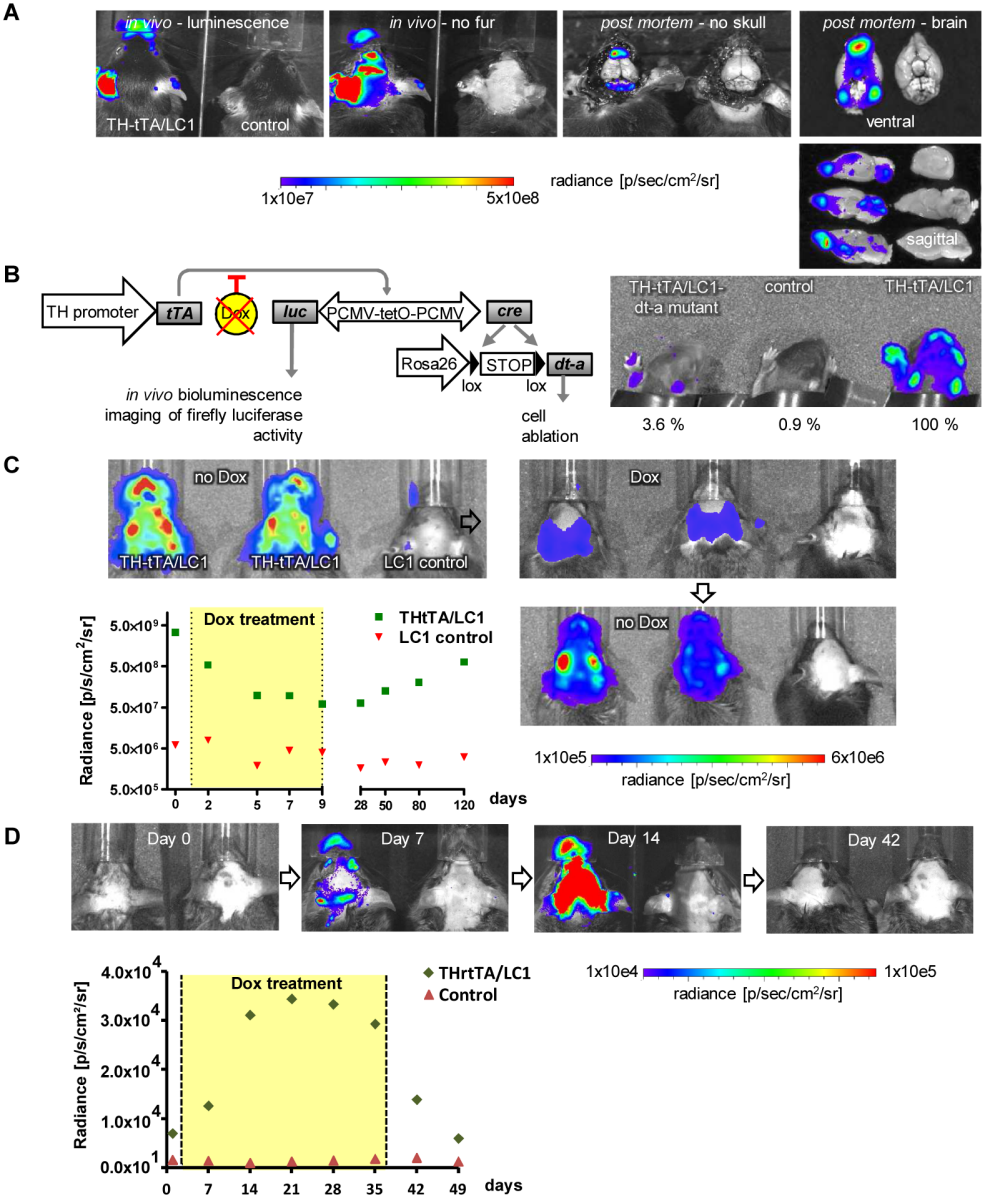
*In vivo* bioluminescence imaging of DA neurons in TH-tTA/LC1 and TH-rtTA/LC1 mice. **(A)** Detection of luciferase activity in anesthetised TH-tTA/LC1 and LC1 control mice after fur removal and in brain tissue of dead mice after removal of the skull and dissection of brains using an IVIS200 *in vivo* imaging device (Calipers Co.). **(B)** Comparison of the *in vivo* bioluminescence signals in P3 TH-tTA/LC1 mouse pups expressing diphteria toxin A (dt-a) with TH-tTA/LC1 control mice and LC1 mice without luciferase expression. The radiance [p/sec/cm2/sr] RAW data luminescence signal intensity is shown in percentage compared to the maximal signal in the TH-tTA/LC1 control mice. **(C)** Temporal control of bioluminescence signal via DOX treatment in TH-tTA/LC1 mice. Quantification of luciferase activity in anesthetised TH-tTA/LC1 and LC1 control mice. After initial measurement, mice were treated with DOX in the drinking water (2 mg/ml) for 8 days and afterwards kept without DOX for further 111 days. Bioluminescence was again measured at the indicated time points, n = 3. **(D)** Temporal control of bioluminescence signal via DOX treatment in TH-rtTA/LC1 mice. Quantification of luciferase activity in anesthetised TH-rtTA/LC1 and LC1 control mice raised on DOX for 6 weeks and kept during adulthood without DOX. After initial measurement, mice were treated with DOX-containing food for 35 days and afterwards kept without DOX for further 14 days, n = 3–8.

**Fig 6:**
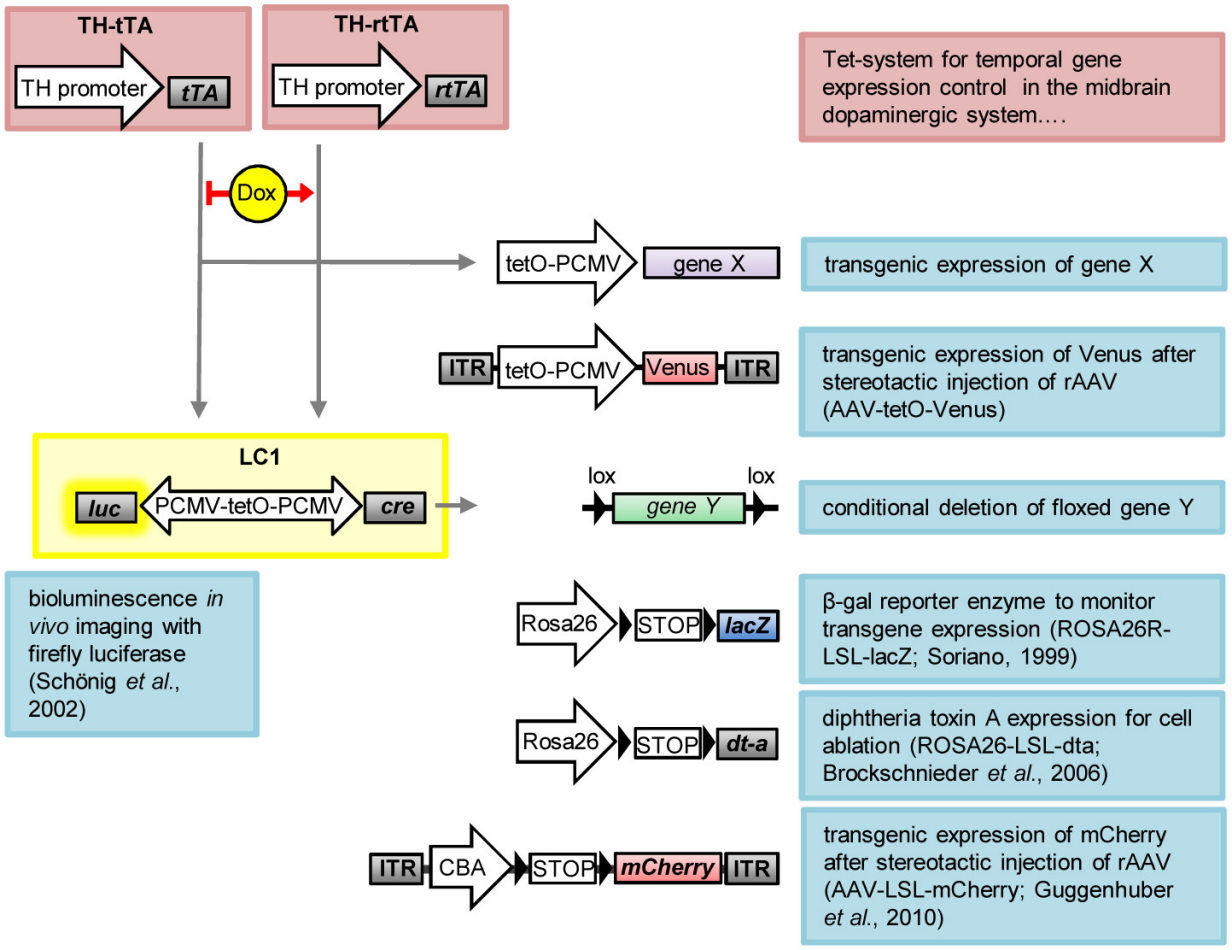
Schematic overview of utilized transgenic mice and recombinant AAV (rAAV) constructs. TH-tTA and TH-rtTA mice express, under the tyrosine hydroxylase (TH) promoter, the tetracycline-regulated transactivator protein (tTA) or the reverse tetracycline-regulated transactivator protein (rtTA) in dopaminergic (DA) neurons. tTA binds to the tetracycline operon (tetO) in the absence of doxycycline (DOX) and rtTA binds in the presence of DOX, driving transient expression of gene X, for example the fluorescent protein Venus from a recombinant AAV vector (AAV-tetO-Venus), or luciferase and Cre from the LC1 construct. The LC1 encodes a luciferase enzyme, which can be used for bioluminescence *in vivo* imaging, and the Cre recombinase for deleting gene Y or removing floxed STOP codons (LSL). Cre is used here as a genetic switch to activate, from the ROSA locus, either expression of the reporter gene lacZ (encoding β-galactosidase) to visualize transgene expression or to activate expression of diphtheria toxin protein A (dt-a) to selectively induce cell death of DA neurons. Here we use Cre also to activate expression of the fluorescent protein mCherry from AAV-LSL-mCherry.

LC1 mice [21], Rosa26R reporter mice [22] and ROSA26-dt-a mice [23] were described previously (Figs 1–6).

### Stereotactic injections of recombinant AAV vectors

The pAAV-tetO-Venus vector was generated by PCR amplification of the tetO sequence fused with the cytomegalovirus (CMV) minimal promoter from pUHD10-3 [24] with primers encoding restriction sites for XbaI 5’ and NheI 3’. The PCR product was cloned into pAAV-hsyn1-Venus [25] substituting the synapsin promoter sequence with the tetO promoter after XbaI and NheI digestion (Fig 2). The AAV-LSL-mcherry DNA using the cytomegalovirus enhancer/chicken beta actin (CBA) promoter was published previously [26].

Before recombinant AAV production, inverted terminal repeat sequences of all rAAV vectors were checked by SmaI digestion. The production of rAAV2/5 vectors was conducted at the Vector Core Facility of the UKE using standard protocols. Typical titers used for *in vivo* transductions in this study were 10^13^ vg ml-1. For substantia nigra injections, glass pipettes were used attached to a 10 μl syringe and an automatic pump at coordinates: bregma: -3.15 mm, lateral: 1.2 mm, ventral: 4.2 mm. A volume of 1 μl virus solution was injected at a rate of 0.2 μl per minute. Injection pipette was left in place for additional 5 minutes before retraction. Adult mice (2–12 months) were maintained after stereotactic injection with the AAV vectors in the ON state for the indicated time points (5 weeks in Fig 2 and 1 to 6 weeks in Fig 4) before fluorescent protein expression was investigated. Some TH-tTA/LC1 mice were treated with DOX-containing food after stereotactic injection as indicated (see Fig 4).

### Histological and immunohistochemical stainings

Mouse brain tissue processing and stainings were conducted as described before [27]. A mouse anti-TH antibody (1:1000; Acris #22941) was used for immunostaining of DA neurons. DAB stainings were mounted in aqueous mounting medium and fluorescent stainings or transgenic sections were mounted in anti-fading reagent (Fluoromount G).

To stain for β-galactosidase activity, sections were first incubated in 0.5% EGTA (v/v), 20 mM MgCl_2_ solution in PBS at room temperature for 15 min and subsequently washed 3 times in washing buffer (2.5 mM MgCl_2_, 0.02% (v/v) Tween 20 in PBS) for 10 min each at room temperature. Sections were stained in X-gal solution in the dark at 37°C for 1 to 24 h depending on the level of β-galactosidase activity. The chromatic reaction was stopped by incubation in 4% PFA on ice for 10 min, followed by washing 3 times in PBS for 10 min at room temperature. Sections were mounted in aqueous mounting medium.

### Conventional microscopy

Transmitted light images were acquired using a SZX16 stereo microscope equipped with a DP72 camera and cellSens Entry 1.4.1 imaging software (Olympus). DAB stained sections and fluorescent cells were imaged with an epifluorescent upright microscope Axio Imager.M1 (Zeiss, Goettingen, Germany) equipped with an automated stage (Ludl MAC 6000 system), Hamamatsu camera C8484 and Axiovision software 4.8. Fluorescent sections were imaged with a Leica TCS SP2 confocal microscope system with 10x (0.3 NA) and 63x (oil 1.32 NA) objectives or Zeiss LSM 700 Confocal microscope system with 10x (0.25 NA) and 40x (oil 1.3 NA) objectives. Confocal images were acquired by using a 514-nm Argon line for Venus or a 561-nm photodiode laser for mCherry. Image stack were maximally projected and contrast and intensity levels were uniformly adjusted using ImageJ (NIH).

### Quantifications

Stereological countings were performed on 30 μm coronal serial sections analyzing every sixth section for the SNpc and VTA. This was done using an oil immersion 63x objective, a counting frame of 50 x 50 μm, and a grid size of 100 x 100 μm, as described previously [27] using the optical fractionator method of the StereoInvestigator software 8.0 (MicroBrightField). To quantify lacZ positive and TH positive cells (Fig 1) we counted at least 2 representative 30 μm serial coronal sections. Venus positive and TH positive cells (Fig 2) were quantified in at least 3 representative 50 μm serial sagittal sections per mouse (every forth section) by applying a binary mask and the “Analyze Particles” function in ImageJ (NIH).

### *In vivo* bioluminescence imaging

Bioluminescence *in vivo* imaging was performed on adult mice (2–12 months in Fig 5A, 5C and 5D) and non DOX treated P3 mice (Fig 5B) by using the IVIS 200 system and Living Image 3.2 software (Perkin Elmer) according to manufacturer´s instructions. For bioluminescence imaging, mice were i.p. injected with D-luciferin aqueous solution (15 mg ml^-1^; 4.5 mg per 30g of bodyweight).

### Statistical analysis

Data are expressed as means + s.e.m. The statistical analyses were performed with GraphPad Prism 5.0 (GraphPad Software) using two-tailed, unpaired Student´s t-test or ANOVA, followed by Tukey‘s post hoc test to compare group means. In all analyses, p-values smaller than 0.05 were considered statistically significant.

## Results

### Generation and basic characterization of TH-tTA and TH-rtTA mice

To develop a tet-OFF and a tet-ON system for DA neurons of the mouse, we cloned the tTA2S or rtTA3G transactivator encoding sequences downstream of an 8.9 kilobase promoter fragment of the mouse TH gene and generated transgenic mice by pronuclear injections (Fig 1; S1 Fig). We generated 12 TH-tTA founder mice of which 9 founders went germline and 9 TH-rtTA founders of which 8 went germline. The founders were functionally characterized by crossing them with LC1 mice [21] and with Rosa26R reporter mice [22] (Fig 1A).

In the triple-transgenic TH-tTA/LC1/Rosa26R mice, the tTA transactivator-induced Cre recombinase expression from the LC1 transgene triggered the deletion of a STOP cassette in front of the LacZ gene, encoding the reporter enzyme β-galactosidase, in the Rosa26 locus. Using X-gal staining, we could detect β-galactosidase activity in midbrain DA neurons in 4 of the 9 TH-tTA founder lines (founder 1 in Fig 1B; founder 2–4 in S2 Fig). 5 founder lines did not show any lacZ expression. The four TH-tTA founder lines showed no difference in the expression pattern without DOX and no obvious physiological or behavioral abnormalities (data not shown).

To switch the tet-system permanently ON in TH-rtTA/LC1/Rosa26R mice, the parents were fed DOX-containing food before pregnancy and their offspring were kept on DOX until analysis. 5 founders showed X-gal staining under these conditions and were similar with regards to expression and physiology (founder 1 in Fig 1C; founder 2–5 in S3 Fig).

To analyze the expression of TH-tTA and TH-rtTA mice in midbrain DA neurons, we performed a co-staining of X-gal with an immunohistochemical TH staining and quantified the double-positive cells. In the TH-tTA/LC1/Rosa26R mice, 54% of substantia nigra pars compacta (SNpc) DA neurons and 58% of ventral tegmental area (VTA) DA neurons were also X-gal labeled (Fig 1D and 1E). TH-rtTA/LC1/Rosa26R mice showed 60% of SN and 65% of VTA DA neurons also positive for X-gal (Fig 1F). Thus, there seems to be an incomplete targeting of midbrain DA neurons in our TH-tet mice, as previously described for other Cre mice using the TH promoter [2–4]. This might be on one hand a disadvantage for systemic approaches, but on the other hand an advantage to enable investigation of targeted and non-targeted DA neurons in the same mice, which is preferred for example in electrophysiological studies.

Besides expression in DA neurons of the VTA and SNpc, DA neurons in the arcuate nuclei and retrorubral field and noradrenergic cells of the locus coeruleus (LC) were stained, too (S4 and S5 Figs). TH expression has been described not only in catecholaminergic neurons, such as DA and noradrenergic neurons, but also transiently or permanent in many other cells inside and outside the nervous system. Transient TH protein expression during development in the absence of catecholamine synthesis has been reported in rodents for example in some interneurons of the cortex [28, 29], mesospiny neurons in the striatum [30] and Purkinje cells of the cerebellum [31]. Also the accessory, deeper layer and periglomerular cells of the olfactory bulb express TH protein during development [1, 32]. However, in adult mice TH protein expression is no longer detectable in these cell populations, although they continue to express TH mRNA [33]. As a consequence, X-gal staining was observed in the TH-tTA/LC1/Rosa26R and TH-rtTA/LC1/Rosa26R mice with the tet-system constantly active in many distinct brain regions such as the cerebellum, pons, striatum, hippocampus, cortex, thalamus, and lateral midbrain (Fig 1B, S4 and S5 Figs). Also, the extent of expression in the different tissues was variable, ranging from activity in all Purkinje cells of the cerebellum and all medium spiny neurons in the striatum to only mosaic, sporadic activation in pyramidal cells in the hippocampus and cortex (S4 and S5 Figs).

In the SNpc and VTA region of TH-tTA/LC1/Rosa26R and TH-rtTA/LC1/Rosa26R mice raised with constitutive active tet-system, more than 60% of X-gal positive cells were also stained with antibodies against TH. Similarly, in transiently DOX treated TH-tTA/LC1/Rosa26R mice, more than 90% of X-gal positive cells were stained (S2B and S3B Figs). Therefore, these cells were considered to be DA neurons. The X-gal-positive cells negative for TH protein expression might be due to cells that only express TH mRNA and no TH protein [34–36] (as described earlier) or cells expressing TH only during development and not adulthood (see next two chapters) (S2B and S2C, S3B and S3C Figs). The endogenous TH enzyme starts to be expressed in the mouse ventral midbrain at embryonic day E9-10 [37, 38] soon after the identification of the first midbrain DA neurons by the expression of the retinoic acid–synthesizing enzyme Aldh1a1 and before DA neurons are functionally connected to their postsynaptic partners [5]. Thus, our TH-tet mice permit targeting of DA neurons very early on during development and requires tight regulation of the tet-system if they are used to alter expression during a later developmental phase, e.g. during adulthood.

### Regulation of gene expression in TH-tTA and TH-rtTA mice

To test the regulation of the tet-system in the TH-tTA and TH-rtTA mice, we first tried to switch off the tet-system completely by constantly feeding TH-tTA/LC1/Rosa26R mice with DOX-containing food or feeding TH-rtTA/LC1/Rosa26R mice with non DOX-containing food. This resulted in very few X-gal positive cells in the ventral midbrain DA system (Fig 1B and 1C). This suggests that there is a tight regulation of the transcriptional activity in both TH-tet mice under these conditions with little leakage.

Next, we wanted to know if we could switch gene expression on or off ad libitum. Therefore, we transiently fed TH-tTA/LC1/Rosa26R mice with DOX until they were 6 weeks of age followed by 6 weeks without DOX before analysis. This protocol enabled us to specifically label X-gal positive DA neurons in the midbrain of 12 week old mice (Fig 1A and 1C, S2B Fig). By applying transient DOX treatment, we targeted in TH-tTA/LC1/Rosa26R mice around 40% of midbrain DA neurons (Fig 1E). To test the tet-system inducibility in TH-rtTA/LC1/Rosa26R mice, we raised them without DOX and started with the DOX treatment at 6 weeks of age for at least 6 additional weeks. However, no X-gal stained cells were observed (Fig 1C). The reduced number of responding DA neurons after DOX treatment in TH-tTA/LC1/Rosa26R mice, as well as the lack of DOX response of adult TH-rtTA/LC1/Rosa26R mice, might be due to genetic silencing of the LC1 construct that prevents re-activation, as previously described [39, 40].

### LC1 independent targeting of adult DA neurons in TH-tTA and TH-rtTA mice

In order to overcome possible genetic silencing of the LC1 construct in TH-rtTA/LC1 mice, we generated an AAV vector expressing the fluorescent protein Venus directly under the control of the tetO promoter (AAV-tetO-Venus). Before virus production, we tested the AAV-tetO-Venus DNA construct in HEK293-T cells co-transfected with a tTA expression vector and found a DOX-dependent expression of Venus (S6 Fig). Subsequently, the AAV-tetO-Venus virus vector was stereotactically injected in the SN of adult non-DOX treated TH-tTA mice and DOX-treated TH-rtTA (Fig 2). We investigated 5 weeks after injection the expression of Venus in the mouse midbrain DA neurons visualized by TH antibody staining. In TH-tTA/AAV-tetO-Venus mice, 67% of DA neurons were also Venus-positive (Fig 2A, 2B and 2E). With DOX treatment 10 days before AAV-tetO-Venus injection, we observed only 27% of Venus-positive DA neurons in TH-tTA mice (Fig 2E, S7 Fig). In DOX-treated TH-rtTA/AAV-tetO-Venus mice, 73% of DA neurons were expressing Venus, while only 23% expressed Venus without DOX (Fig 2C–2E, S7 Fig). In non-transgenic control mice, AAV-tetO-Venus injection resulted in only 5% of Venus-positive DA neurons, which was comparable with background expression seen in AAV-tetO-Venus transfected HEK293-T cells (Fig 2E, S6 and S7 Figs). This result confirms that it is possible to induce the tet-system in TH-rtTA mice with a transient postnatal DOX treatment. Furthermore, it provides evidence that in both transgenic mouse lines, the TH-tTA and TH-rtTA mice, the tet-system can efficiently activate expression from virally provided and tetO regulated genes of interest during adulthood.

### Genetic deletion of DA neurons in TH-tTA mice

We made use of our TH-tTA mice to generate a genetic mouse model with reduced number of DA neurons. To do this, we replaced the Rosa26R with the Rosa26dt-a transgene in the triple-transgenic mice that, in the absence of DOX, induced cell death by the expression of diphtheria toxin-A [23] (Fig 3A). TH-tTA/LC1/Rosa26dt-a mice without DOX died shortly after birth in the first postnatal week and lost around 40% of midbrain DA neurons in the SNpc and VTA (Fig 3B and 3C).

The mice survived until adulthood if transiently treated with DOX during embryogenesis and early postnatal development until 6 weeks of age. We stereologically quantified the number of DA neurons in mice 6 weeks and between 14 to 30 weeks after removal of DOX and found 18 to 32% less DA neurons in the SNpc and 28 to 33% less DA neurons in the VTA, respectively (Fig 3D and 3E). This supports the tight regulation of the tet-system in our TH-tTA mice and the possibility to generate, with this approach, a genetic adult-onset DA system neurodegeneration mouse model.

### Fluorescent labeling of adult DA neurons in TH-tTA mice

Our next goal was to test the TH-tet mice for developing an efficient system for fluorescently labeling DA neuron cell bodies and axons for fast assessment of the DA fiber density in the striatum and possibly axonal tracing experiments. The currently available fluorescent DA neuron marker mice, such as the TH-GFP [41, 42], Pitx3-GFP [43] or DAT-GFP [44] mice efficiently label the DA cell bodies, but only weakly label the DA innervation in the target fields. This includes the striatum, which is important to investigate in the DA system degeneration or regeneration experiments related to PD. This is most likely due to the low copy number of fluorescent protein encoding cDNAs in the transgenic mice and the very long and manifold brunched axons of DA neurons.

Since the stereotactic injection of the AAV-tetO-Venus vector in the TH-tTA and TH-rtTA mice did not efficiently label the complete axonal network of DA neurons, we tried to enhance the fluorescent signal by using the Cre-lox system. We stereotactically injected adult TH-tTA/LC1 mice in the SNpc with an AAV-LSL-mCherry virus construct, that uses the strong cytomegalovirus enhancer/chicken beta actin (CAG) promoter followed by a floxed STOP codon (LSL) and the mCherry expression cassette [26] (Fig 4A). As an alternative DA neuron-specific Cre mouse line, we also injected DAT-Cre mice with the same AAV-LSL-mCherry virus [7] (Fig 4A). In both mouse lines the STOP codon was efficiently removed in midbrain DA neurons and allowed to label them with mCherry (Fig 4B–4O). To determine the minimal time required to label DA axons in the striatum, we analyzed the mice 1, 2, 3, 4, 5 and 6 weeks after virus injection. We found that 1 week was sufficient to label a few cell bodies, but after 5 weeks there was a strong fluorescent staining of midbrain DA neurons and of the DA fibers in the striatum for both AAV-LSL-mCherry injected mouse lines, DAT-Cre (Fig 4B–4G) and TH-tTA/LC1 mice (Fig 4H, 4I, 4L and 4M). In both Cre lines injected with AAV-LSL-mCherry, DAT-Cre and TH-tTA/LC1 mice, we did not observe labeled fibers in the lateral habenula (LHb), which is still an open question in the field (Fig 4P and 4Q) [34–36, 45, 46]. Treating the TH-tTA/LC1 mice after viral injection for 4 or 6 weeks with DOX strongly reduced the amount of labeled cells in the midbrain and the fiber staining in the striatum (Fig 4J, 4K, 4N and 4O). Together with the previous experiments this shows the tight regulation of the tet-system in our TH-tTA mice during adulthood. These data suggests that the combination of TH-tTA/LC1 mice with the AAV-LSL-mCherry virus is an excellent approach to efficiently label TH expressing neurons and their axons in their target fields such as the striatum.

### In vivo bioluminescence imaging of the DA system in TH-tTA and TH-rtTA mice

To be able to visualize and monitor the DA system in living mice over time, we made use of luciferase expression from the LC1 construct in our TH-tTA/LC1 mice. Systemic application of the D-luciferin substrate allowed us to detect a bioluminescence signal through all openings of the skull, the nose, ears and eyes of the double transgenic mice, but no signal in single transgenic mice (Fig 5A). Removing the black fur improved the detection of the *in vivo* signal through the skull directly above the brain (Fig 5A). Removing the skin and skull post mortem enabled us to detect the luminescence signal directly above the specific brain regions with TH expression, such as the olfactory bulb, the midbrain and the cerebellum, without further scattering of the signal (Fig 5A). Further dissection of the brain showed that the bioluminescence signal arises from the TH-positive cells already detected in the TH-tTA/LC1/ROSA26R mice with lacZ staining (Fig 1). The specificity of the *in vivo* signal was confirmed in TH-tTA/LC1/Rosa26dt-a mice, in which the signal was strongly diminished (Fig 5B). This is consistent with the view that almost all the cells, in which LC1 was active and led to luciferase and Cre expression, died due to the Cre-mediated diphtheria toxin-A expression. Treating shaved TH-tTA/LC1 mice for 8 days with DOX-containing food continuously reduced the *in vivo* bioluminescence signal, consistent with a down regulation of luciferase in the mice (Fig 5C). Subsequently, the bioluminescence signal increased slowly again when the mice were on DOX-free food (Fig 5C). In TH-rtTA/LC1 mice raised on DOX for 6 weeks and maintained without DOX during adulthood, we could show reversible gene activation during adulthood with faster ON-kinetics compared to the TH-tTA/LC1 mice (Fig 5D). TH-rtTA/LC1 mice without DOX showed only a very weak luciferase signal that increased after 2 weeks on DOX to the maximal luciferase signal and decreased again to the original levels 2 weeks after withdrawal from DOX (Fig 5D).

Thus, the bioluminescence signal in TH-tTA/LC1 and TH-rtTA/LC1 mice allows *in vivo* monitoring of the integrity and gene expression activity of the DA system, and might therefore be applied to follow degeneration and regeneration processes of the DA system over time in the same mouse.

## Discussion

The TH-tTA and TH-rtTA mouse lines described enable targeting of midbrain DA neurons with high efficiency during all developmental stages, show a tight DOX-dependent regulation and therefore can be used for a multitude of applications (Fig 6). We have shown that the TH-tTA and TH-rtTA mice are an excellent tool for regulating gene expression in midbrain DA neurons. They can be used to overexpress or delete genes of interest and they can be combined with the Cre-lox recombination system (Figs 1–6). Therefore, our TH-tTA and TH-rtTA mouse lines permit studying, for example, the loss of function phenotype of genes in the adult DA system that lead to embryonic lethality or possible activation of compensatory mechanisms during development, which might mask their function during adulthood. In addition, we show that the TH-tet mice can be efficiently used to visualize DA neurons and their innervation fluorescently in the brain (Figs 2 and 4). Furthermore, they can be used to monitor the DA system *in vivo* as an entity over the whole life time of a mouse and therefore allow following alterations progressively over time (Fig 5).

In 2004, the first ratTH-rtTA2S-M2 construct was generated and successfully used in HEK293 cells and in the dopaminergic cell line MN9D [47]. However, three groups have only recently generated transgenic mice in which the tet-system was targeted to midbrain DA neurons [48, 49]. In the first mouse line, a tTA knock-in was created into the endogenous DAT locus, which generates a heterozygote DAT knockout mouse showing low DAT levels and hyperactivity [50]. In a second line, a TH-rtTA mouse line, tetracycline-dependent regulation was subsequently lost [49](data not shown). In the third mouse line, carrying a Pitx3-tTA construct, tetracycline-dependent regulation has not yet been shown [48]. To preserve our functional tet-system mouse lines for the research community, we immediately conserved them by sperm freezing.

The TH-tTA and TH-rtTA mice can be combined with many different tetO-constructs at the same time and used to activate or inhibit gene expression during embryogenesis, adulthood and in aging mice since DOX is rapidly taken up into cells, shows low toxicity and penetrates the placenta and the blood-brain barrier [51–53]. Silencing of tetO-constructs in the mouse can be overcome, as shown here, by administering it at specific time points, for example, by electroporation of DNA in embryos or by viral injections in adult mice. If a gene should only be expressed for a short time period, the rapid inducible tet-ON system might be preferred. However, if a gene should only be switched off for a short time period the use of the tet-OFF system might be an advantage and can save DOX-food costs. In order to decide between each system, it is worthwhile mentioning that the tet-OFF systems shows a slower ON-kinetics (several weeks) compared to the tet-ON system (several days), as shown also in our *in vivo* luciferase experiment. Furthermore, long DOX-treatment times might lead to an accumulation of DOX in the bone that is only slowly cleared after removal of DOX from the food or water [51–53].

The mosaic or incomplete targeting of DA neurons in our mouse TH promoter-driven system seems to be a common issue and has already been reported for transgenic mice generated with the rat TH promoter [2, 4]. This seems to be the case even in mice with the transgene knocked into the endogenous mouse TH locus behind an internal ribosomal entry sequence (IRES) [3]. Besides the TH promoter specific features this appears to be at least in part also due to the reporter construct used as viral vectors and transgenic mice do not express their encoded reporter homogeneously in DA neurons [3]. In addition, we did not find any fluorescently labeled DA neurons when we crossed the TH-tTA/LC1 mice with Ai9 tdTomato reporter mice [54] (data not shown). This is surprising, because Ai9 mice use like the Rosa26R and Rosa26dt-a mice the Rosa locus for tdTomato expression [22]. Yet, Ai9 mice labeled DA neurons nicely when crossed with DAT-Cre mice [54](data not shown). Despite this, more research is needed to better understand the underlying biology of this phenomenon. The mosaic targeting can also be an advantage for some experiments. In studies where DA cells with different genetic alterations need to be compared *in vivo* in the same environment—for example, in electrophysiological studies—the partial targeting of DA neurons in the TH-tTA mice will turn out to be a very helpful condition, much like the MARCM clones in *Drosophila melanogaster* that turned out to be an excellent tool to study *in vivo* gene function in the wildtype background of flies [55]. The mosaic expression in the TH-tTA/LC1 mice allowed us to establish viable TH-tTA/LC1/Rosa26dt-a mice showing a very reproducible and progressive DA system degeneration in young adult mice reaching in 3–7 month old mice 30% of DA neuron loss. The DA system degeneration in TH-tTA/LC1/Rosa26dt-a mice is less variable and cumbersome to generate compared to the commonly used injections of neurotoxins such as MPTP, 6-OHDA or rotenone to generate PD animal models [56]. Therefore, the inducible DA system degeneration phenotype in TH-tTA/LC1/Rosa26dt-a mice might be a favorable alternative to test cell and tissue replacement therapies for the treatment of PD.

The neonatal lethality in TH-tTA/LC1/Rosa26dt-a mice is most likely due to the loss of noradrenergic neurons in the peripheral nervous system innervating for example the heart. This view is supported by the findings that DAT-Cre/Rosa26dt-a mice also show a reduced number of midbrain DA neurons but are viable and show a normal motor behavior despite the DA cell loss of around 90% [57]. In addition, TH deficiency in mice has been shown to be embryonal lethal apparently due to cardiovascular failure [58] and the lethality can be rescued by knocking-in TH into the noradrenergic-specific dopamine-β-hydroxylase locus [59]. DOX treatment of TH-tTA/LC1/Rosa26dt-a mice during development prevents postnatal lethality and allows deleting dopaminergic neurons during adulthood.

To enable the unbiased fluorescent detection of DA neuron axonal branches and to allow *in vivo* detection of the fluorescently labeled DA system in our TH-tet mice, we made use of a Cre-mediated expression of mCherry from an AAV vector (AAV-LSL-mCherry). We show as a proof of principle that for this purpose the AAV5-LSL-mCherry virus can be used in combination with both mouse lines expressing Cre recombinase in DA neurons, the DAT-Cre and the TH-tTA/LC1 mice. The advantage of the TH-tTA/LC1 mice becomes apparent if you want to label DA neurons in which genetic alterations should not only be induced by Cre recombinase events but also by the tet-O promoter enabling reversible gene activation.

Recently, the approach to characterize in more detail the VTA cell population innervating the LHb to promote reward led to the finding that different TH and DAT promoter driven Cre mice label in combination with different AAV viruses, distinct VTA neurons [34–36, 45, 46]. In summary, three different TH promoter driven Cre lines seem to have targeted neurons which innervate the LHb, express TH, GAD and VGlut2 mRNA [46], but do not express TH protein, and have electrophysiological properties like GABAergic and glutamatergic neurons. In addition, one identical DAT-Cre mouse line was used by two groups in combination with two different AAV reporter constructs. One group reported targeting of VTA neurons innervating the LHb, the other group not [35, 36]. We have detected no DA innervation of the LHb from the midbrain with our AAV-LSL-mCherry virus in the same DAT-Cre mouse line used before and in our TH-tTA/LC1 mice. Taken together these results support the idea that not only the Cre expressing mouse line but also the transgenic or viral reporter construct dramatically influences the outcome of targeting and that the outcome of a specific combination of Cre and reporter is so far not predictable.

We can imagine that combining several AAV-LSL-XFP viruses encoding different fluorescent proteins (XFP) might allow labeling and tracing single DA neurons in almost unique colors. This could facilitate the detailed analysis of the heterogeneous population of DA neurons [1, 60] for which only 8 neurons have been traced in detail from the rat brain and none from the mouse brain [61]. Further experiments are needed to test this hypothesis in detail.

We provide here the proof of principle that *in vivo* imaging of the DA system in our TH-tet mice is feasible using bioluminescence signals. *In vivo* imaging might allow detecting progressive degeneration and regeneration processes of the DA system over time in individual mice and therefore might be suitable for testing drugs for their beneficial or detrimental effect. This method should allow cutting down the number of animals in longitudinal studies and should avoid inter-subject variability problems. Since the *in vivo* measurement takes only seconds to minutes, it might reduce the time for analysis dramatically and can replace or precede a detailed histological analysis. The special resolution and signal intensity of the *in vivo* signal might be further improved by replacing the skull with a glass plate, using more sensitive bioluminescence substrates such as Cycluc1 [62], and by switching for bioluminescence imaging to a red-shifted luciferase variant [63].

In the future, TH-tet mice might also be used for altering and monitoring acute DA cell physiology by combining them with Ca2+ imaging and optogenetic tools, such as in AAV-LSL-channelrhodopsin or halorhodopsin vectors and the KENGE-tet system, using knockin-mediated enhanced gene expression by improved tetracycline-controlled gene induction to express a highly light-sensitive channelrhodopsin-2 mutant at levels sufficient to drive the activities in different cells [64, 65].

With the TH-tTA and TH-rtTA mice we generated a versatile tet-system available to genetically modify the DA system of the mouse for studying *in vitro* and *in vivo* DA neuron development and maintenance, physiology and pathophysiology, and biochemical or histological alterations. The establishment of the tet-OFF and tet-ON system in mouse DA neurons enlarged our tool kit for more sophisticated and fine-tuned manipulations of the DA system that should help to increase our knowledge about this complex and fascinating system and might shed more light on the etiology and possible treatments of human diseases such as PD, schizophrenia and drug addiction.

## Supporting Information

S1 Fig Schematic overview of transgenic TH-tTA mice. **(A)** Scheme of the TH-tTA DNA construct: transcription of the tetracycline transactivator protein tTA is controlled by a 8.9 kb mouse genomic fragment upstream of the AUG start codon of the tyrosine hydroxylase (TH) coding sequence. **(B)** Agarose gel analysis of the TH-tTA fragment isolated from the SalI cut plasmid used for pronucleus injection (PI) into C57Bl/6J single cell embryos to generate transgenic mice. 1: GeneRuler 1kb DNA ladder; 2: TH-tTA DNA fragment of around 12 kb. **(C)** Principle of the Tet-OFF system: tTA protein is expressed via the TH-tTA construct and binds in the absence of doxycycline (DOX) to the tet-responsive promoter (*tetO*) and activates gene of interest expression.
(TIF)

S2 Fig Specificity, inducibility and efficacy of gene expression in additional TH-tTA founders after crossing them with LC1 and Rosa26R mice. **(A)** Coronal midbrain brain sections with SNpc and VTA DA neurons of TH-tTA/LC1/Rosa26R for founders 2, 3, and 4, respectively. Mice were raised without (no) DOX, with (constant) DOX or with DOX until the age of 6 weeks followed by 6 weeks without (transient) DOX. Sections were stained for beta-galactosidase activity with X-gal to visualize cells with activated tTA, LC1 and ROSA26 locus. Scale bars: 500 μm. **(B)** Ratio of lacZ and TH double positive cells to all lacZ positive cells in the SNpc and VTA in TH-tTA/LC1/Rosa26R without or transient DOX treatment as indicated. **(C)** lacZ positive cells in the VTA, SNpc and ventral midbrain without VTA and SNpc in non DOX treated TH-tTA/LC1/Rosa26R mice.
(TIF)

S3 Fig Specificity, inducibility and efficacy of gene expression in additional TH-rtTA founders after crossing them with LC1 and Rosa26R mice. **(A)** Coronal midbrain brain sections with SNpc and VTA DA neurons of TH-rtTA/LC1/Rosa26R for founders 2, 3, 4, and 5, respectively. Mice were raised with (constant) DOX, without (no) DOX, or transiently without DOX until the age of 6 weeks followed by 6 weeks with (transient) DOX. Sections were stained for beta-galactosidase activity with X-gal to visualize cells with activated rtTA, LC1 and ROSA26 locus. Scale bars: 500 μm. **(B)** Ratio of lacZ and TH double positive cells to all lacZ positive cells in the SNpc and VTA in TH-rtTA/LC1/Rosa26R with constant DOX treatment. **(C)** lacZ positive cells in the VTA, SNpc and ventral midbrain without VTA and SNpc in constantly DOX treated TH-rtTA/LC1/Rosa26R mice.
(TIF)

S4 Fig Specificity, inducibility and efficacy of gene expression outside midbrain DA neurons in adult mice of TH-tTA founder 1 after crossing with LC1 and Rosa26R mice. Coronal mouse brain sections stained with X-gal to visualize β-galactosidase expression in TH-tTA/LC1/Rosa26R mice without (A,C, E, G) or transient DOX (B, D, F, H). Broad lacZ expression was detected in mice without DOX treatment in the cerebellum and pons (A), in the striatum (C), the olfactory bulb (E), the hippocampus and different layers of the cortex (G). Mice transiently treated with Dox during development till 6 weeks after birth and evaluated 6 weeks later showed still X-gal staining in the cerebellum (B) but not anymore in the pons (B), the striatum (D), hippocampus and cortex (H). Scale bars: 200 μm (A-H), blowup 500 μm (A-D). **(I)** Quantification of TH+ lacZ+ double positive cells in the locus coeruleus of TH-tTA/LC1/Rosa26R mice without DOX revealed 17% targeted noradrenergic neurons.
(TIF)

S5 Fig Specificity and efficacy of gene expression outside midbrain DA neurons in adult mice of TH-rtTA founder 1 after crossing with LC1 and Rosa26R mice. Coronal mouse brain sections stained with X-gal to visualize β-galactosidase expression in TH-rtTA/LC1/Rosa26R mice with DOX. Broad lacZ expression was detected in the cerebellum and pons (A), hippocampus (B), striatum (C), in different layers of the cortex (B and C) and the olfactory bulb (D). Scale bars: 200 μm (A and C), blowup 500 μm (A-D).
(TIF)

S6 Fig Transfection of new pAAV-tetO-Venus DNA construct in HEK293-T cells. Cells were transfected with the following DNA constructs and analyzed 48 hours later for Venus expression: **(A)** pUHD61-1-tTA vector encoding tTA under a CMV promoter; **(B)** pAAV-tetO-Venus vector encoding Venus under the tetO promoter; and **(C)** co-transfection of both pUHD61-1-tTA and pAAV-tetO-Venus vector. Scale bar: 200 μm.
(TIF)

S7 Fig Cre-independent adult gene expression in DA neurons of TH-tTA and TH-rtTA mice using a AAV-tetO-Venus vector. **(A-C)** Confocal fluorescent pictures of Venus expression (green) in DA neurons co-stained for TH (red) in the substantia nigra pars compacta (SNpc) of sagittal mouse brain sections 5 weeks after AAV-tetO-Venus virus injection. TH-tTA mice were treated with DOX for 10 days before streotactic injections until analysis to switch the system off (A). TH-rtTA mice were here not DOX treated to keep the system inactive (B). Non-transgenic control mice were analyzed also 5 weeks after AAV-tetO-Venus virus injection (C). Scale bars: 250 μm.
(TIF)
