# Supplementary figures and images for: An Efficient and Versatile System for Visualization and Genetic Modification of Dopaminergic Neurons in Transgenic Mice
Source: PLoS One. 2015 Aug 20;10(8):e0136203. doi: 10.1371/journal.pone.0136203 (PMC4546329; doi:10.1371/journal.pone.0136203)

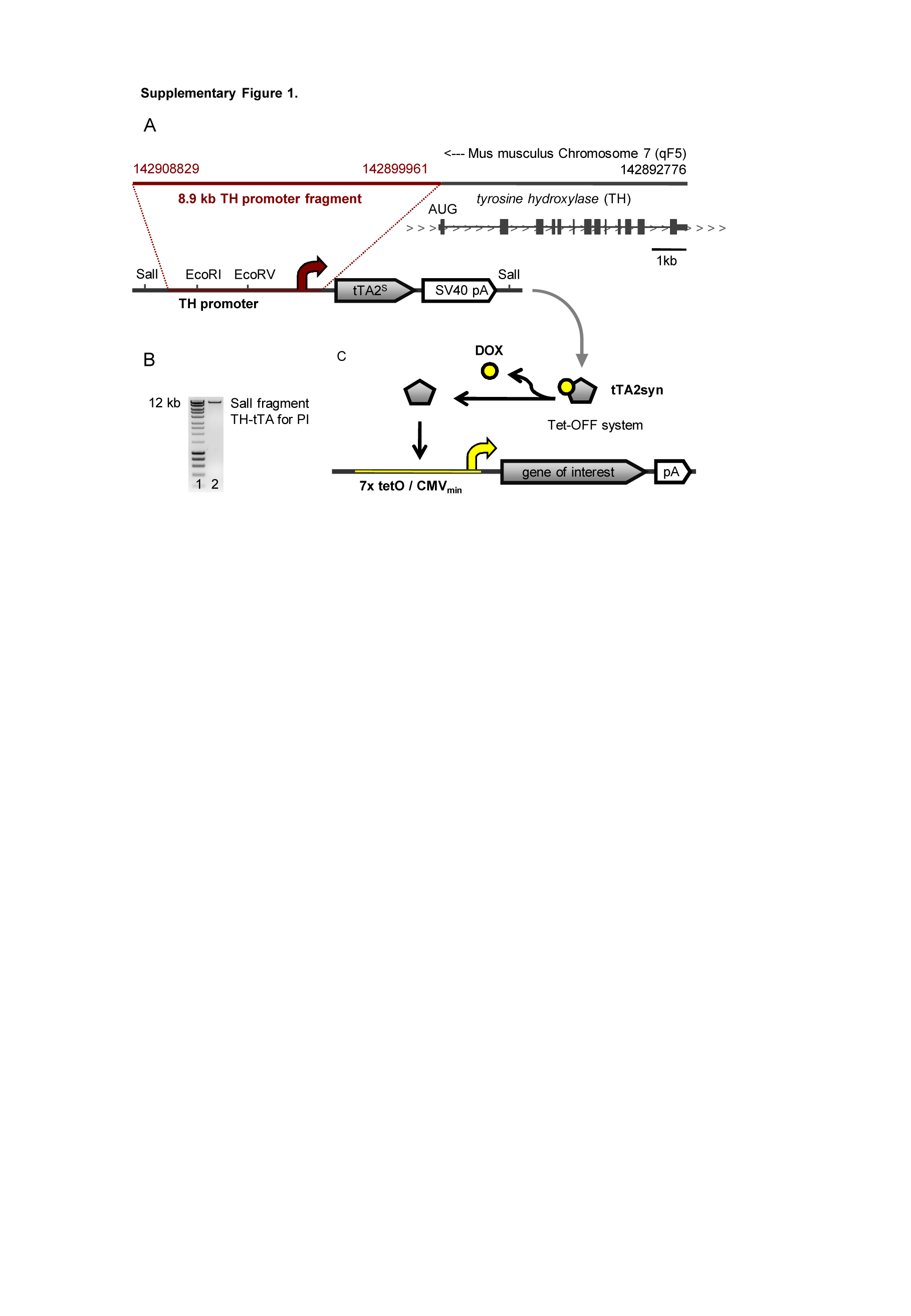

Supplement: S1 Fig — (A) Scheme of the TH-tTA DNA construct: transcription of the tetracycline transactivator protein tTA is controlled by a 8.9 kb mouse genomic fragment upstream of the AUG start codon of the tyrosine hydroxylase (TH) coding sequence. (B) Agarose gel analysis of the TH-tTA fragment isolated from the SalI cut plasmid used for pronucleus injection (PI) into C57Bl/6J single cell embryos to generate transgenic mice. 1: GeneRuler 1kb DNA ladder; 2: TH-tTA DNA fragment of around 12 kb. (C) Principle of the Tet-OFF system: tTA protein is expressed via the TH-tTA construct and binds in the absence of doxycycline (DOX) to the tet-responsive promoter (tetO) and activates gene of interest expression. (TIF) [file pone.0136203.s001.tif]

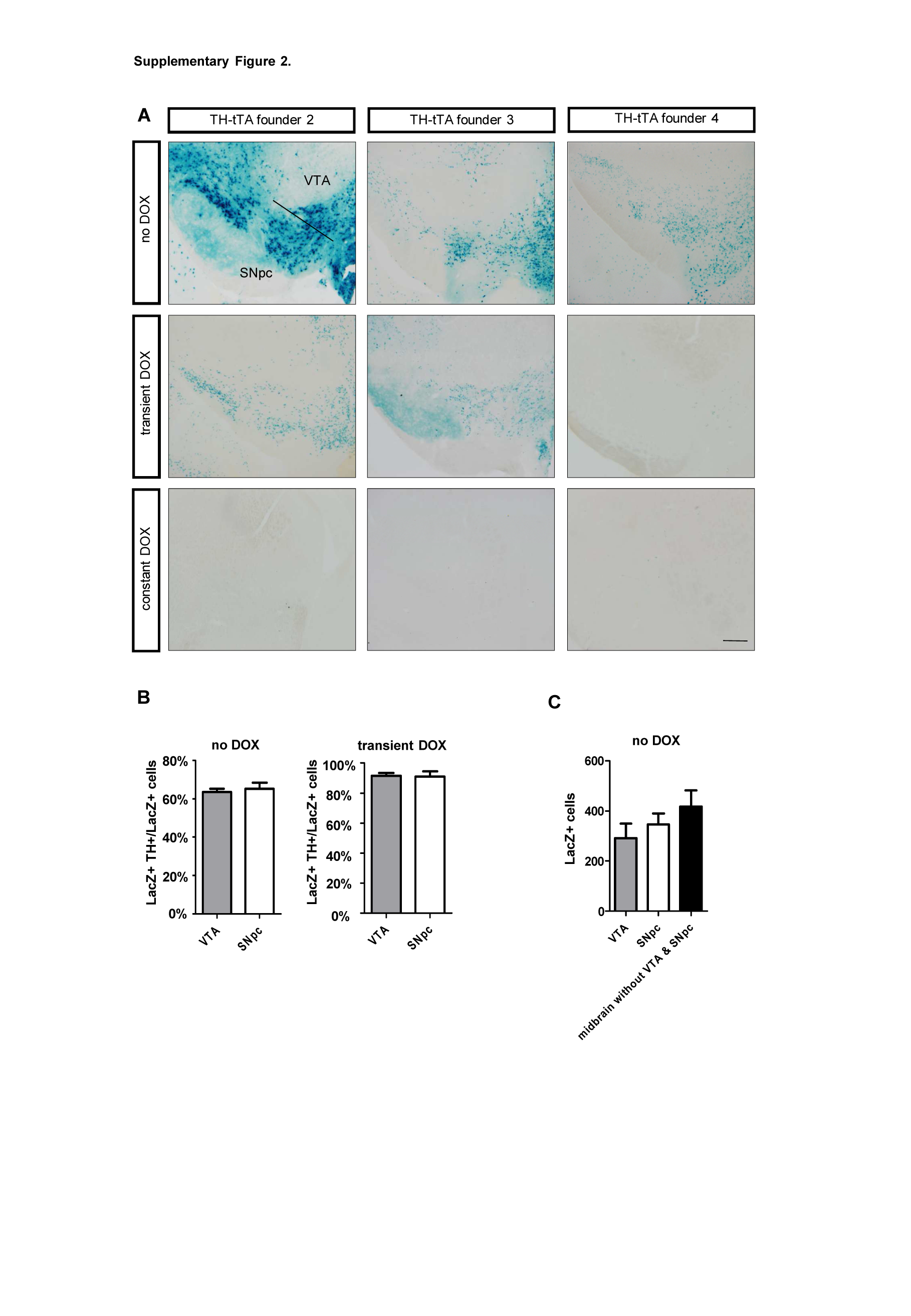

Supplement: S2 Fig — (A) Coronal midbrain brain sections with SNpc and VTA DA neurons of TH-tTA/LC1/Rosa26R for founders 2, 3, and 4, respectively. Mice were raised without (no) DOX, with (constant) DOX or with DOX until the age of 6 weeks followed by 6 weeks without (transient) DOX. Sections were stained for beta-galactosidase activity with X-gal to visualize cells with activated tTA, LC1 and ROSA26 locus. Scale bars: 500 μm. (B) Ratio of lacZ and TH double positive cells to all lacZ positive cells in the SNpc and VTA in TH-tTA/LC1/Rosa26R without or transient DOX treatment as indicated. (C) lacZ positive cells in the VTA, SNpc and ventral midbrain without VTA and SNpc in non DOX treated TH-tTA/LC1/Rosa26R mice. (TIF) [file pone.0136203.s002.tif]

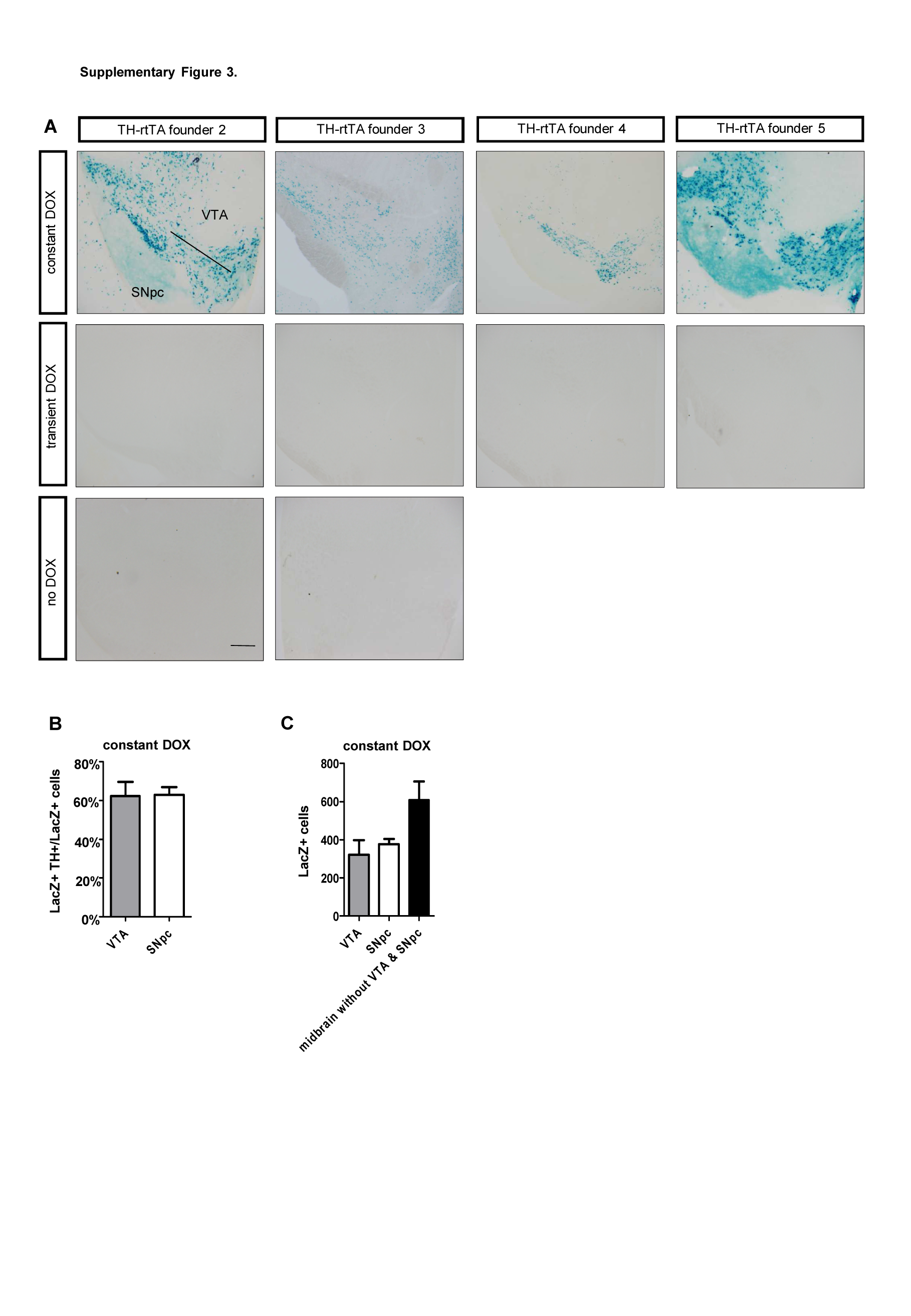

Supplement: S3 Fig — (A) Coronal midbrain brain sections with SNpc and VTA DA neurons of TH-rtTA/LC1/Rosa26R for founders 2, 3, 4, and 5, respectively. Mice were raised with (constant) DOX, without (no) DOX, or transiently without DOX until the age of 6 weeks followed by 6 weeks with (transient) DOX. Sections were stained for beta-galactosidase activity with X-gal to visualize cells with activated rtTA, LC1 and ROSA26 locus. Scale bars: 500 μm. (B) Ratio of lacZ and TH double positive cells to all lacZ positive cells in the SNpc and VTA in TH-rtTA/LC1/Rosa26R with constant DOX treatment. (C) lacZ positive cells in the VTA, SNpc and ventral midbrain without VTA and SNpc in constantly DOX treated TH-rtTA/LC1/Rosa26R mice. (TIF) [file pone.0136203.s003.tif]

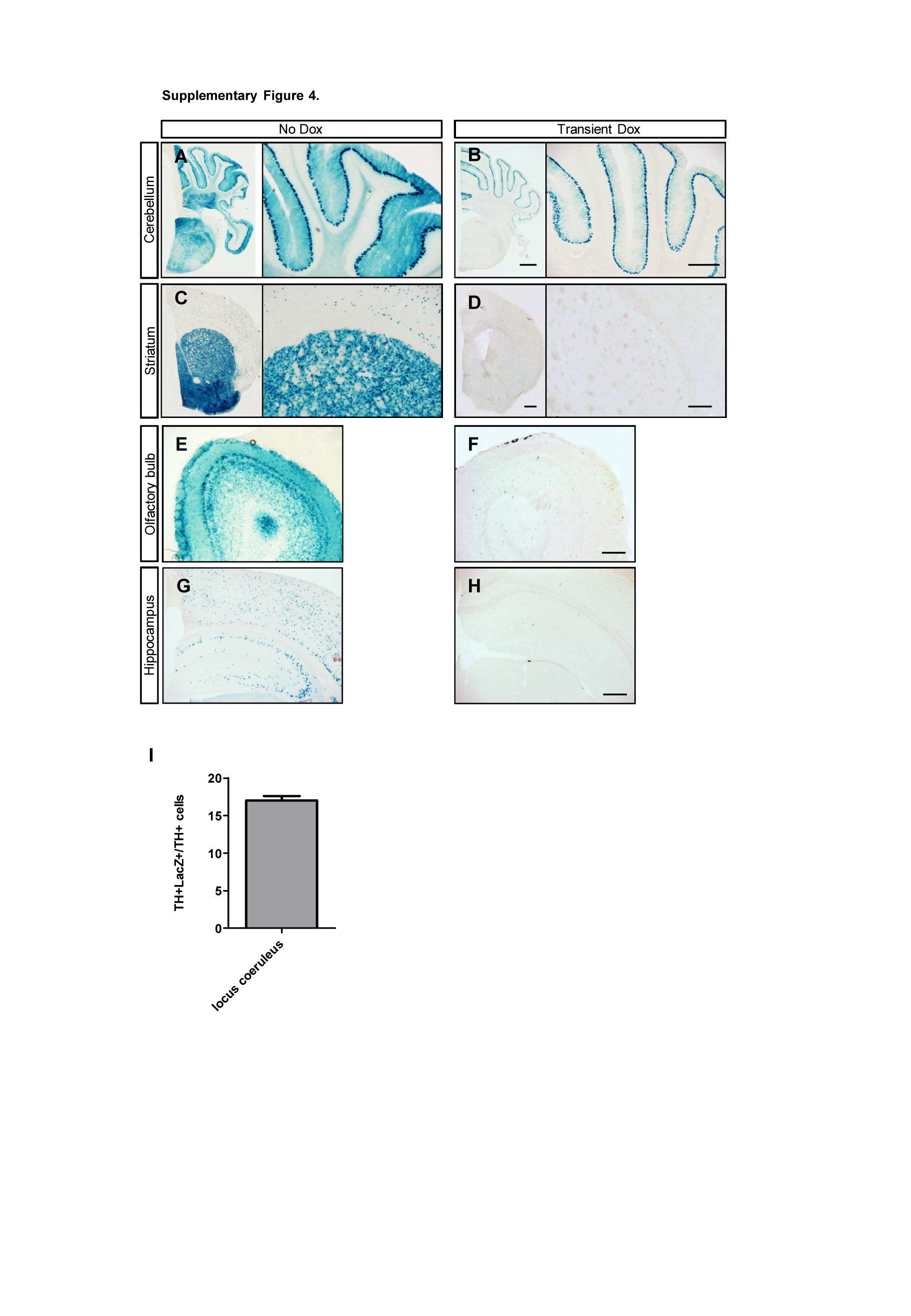

Supplement: S4 Fig — Coronal mouse brain sections stained with X-gal to visualize β-galactosidase expression in TH-tTA/LC1/Rosa26R mice without (A,C, E, G) or transient DOX (B, D, F, H). Broad lacZ expression was detected in mice without DOX treatment in the cerebellum and pons (A), in the striatum (C), the olfactory bulb (E), the hippocampus and different layers of the cortex (G). Mice transiently treated with Dox during development till 6 weeks after birth and evaluated 6 weeks later showed still X-gal staining in the cerebellum (B) but not anymore in the pons (B), the striatum (D), hippocampus and cortex (H). Scale bars: 200 μm (A-H), blowup 500 μm (A-D). (I) Quantification of TH+ lacZ+ double positive cells in the locus coeruleus of TH-tTA/LC1/Rosa26R mice without DOX revealed 17% targeted noradrenergic neurons. (TIF) [file pone.0136203.s004.tif]

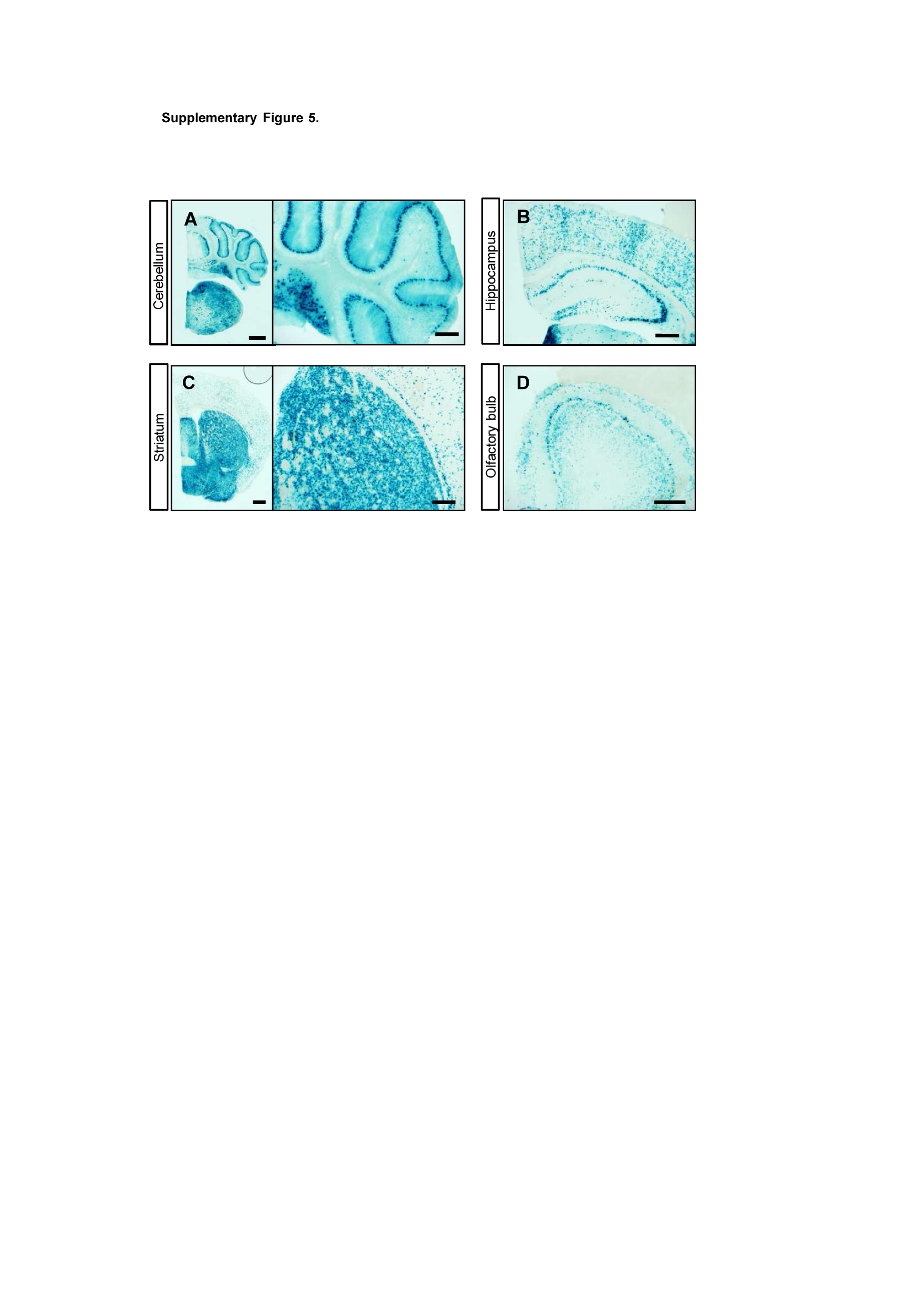

Supplement: S5 Fig — Coronal mouse brain sections stained with X-gal to visualize β-galactosidase expression in TH-rtTA/LC1/Rosa26R mice with DOX. Broad lacZ expression was detected in the cerebellum and pons (A), hippocampus (B), striatum (C), in different layers of the cortex (B and C) and the olfactory bulb (D). Scale bars: 200 μm (A and C), blowup 500 μm (A-D). (TIF) [file pone.0136203.s005.tif]

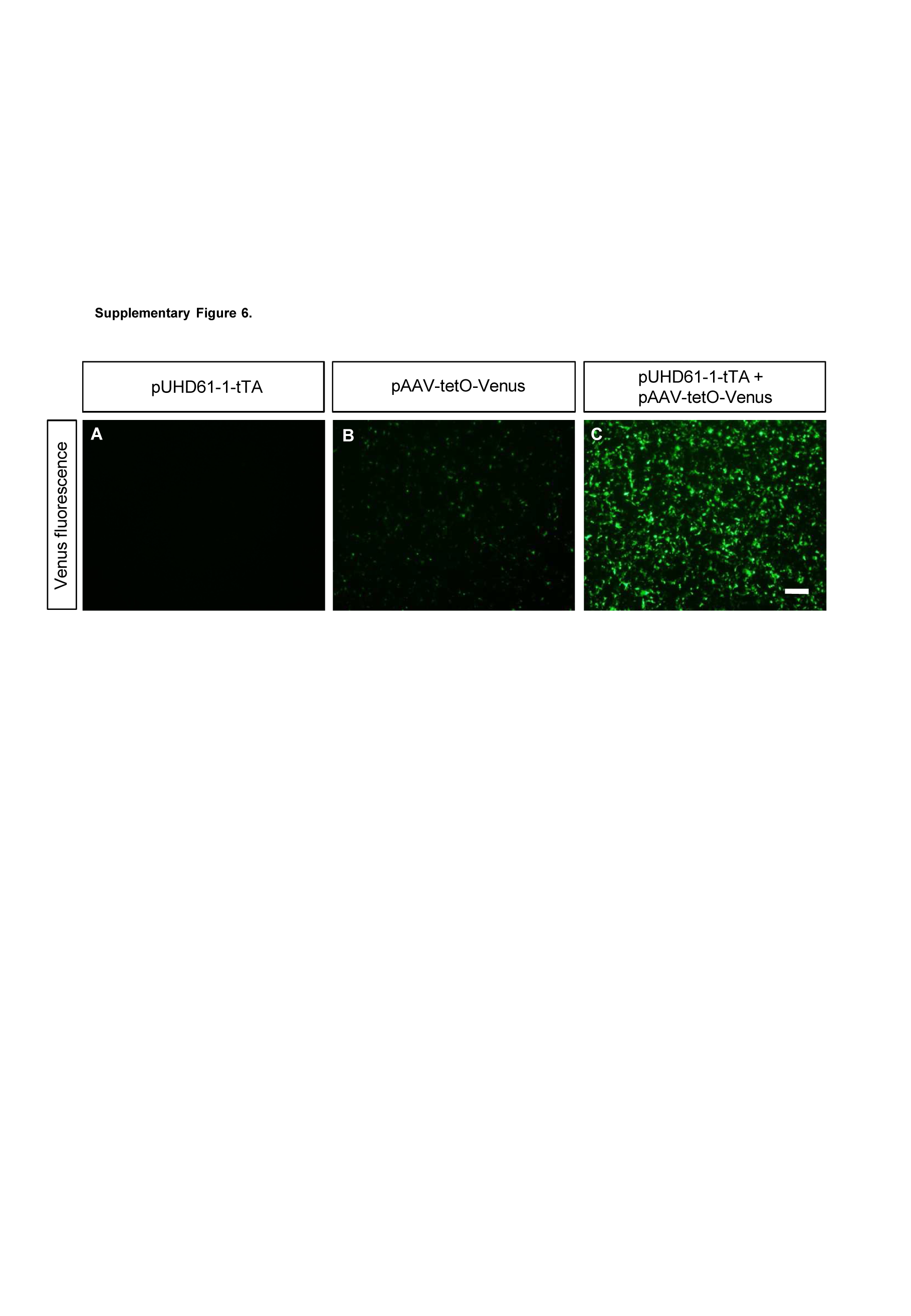

Supplement: S6 Fig — Cells were transfected with the following DNA constructs and analyzed 48 hours later for Venus expression: (A) pUHD61-1-tTA vector encoding tTA under a CMV promoter; (B) pAAV-tetO-Venus vector encoding Venus under the tetO promoter; and (C) co-transfection of both pUHD61-1-tTA and pAAV-tetO-Venus vector. Scale bar: 200 μm. (TIF) [file pone.0136203.s006.tif]

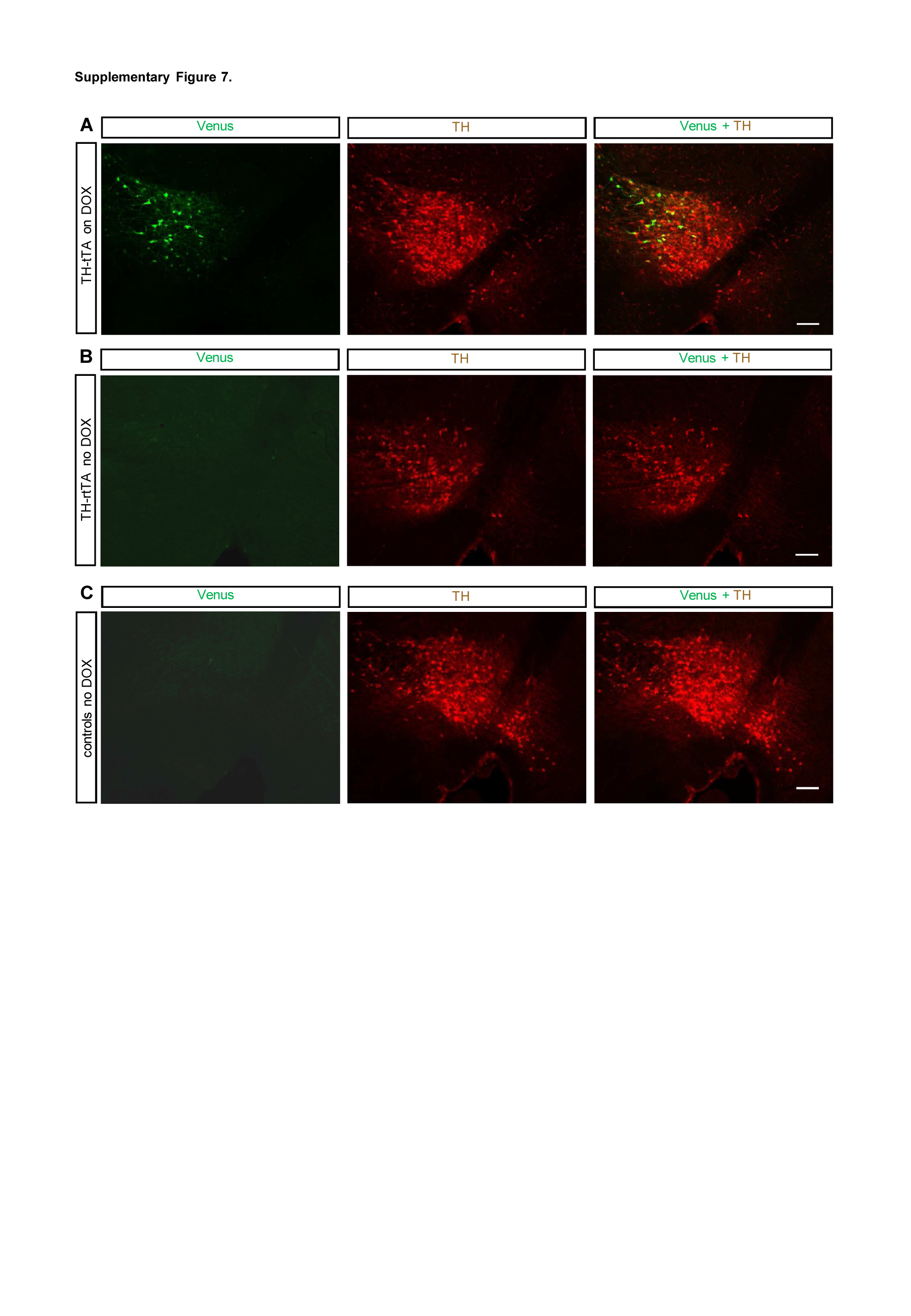

Supplement: S7 Fig — (A-C) Confocal fluorescent pictures of Venus expression (green) in DA neurons co-stained for TH (red) in the substantia nigra pars compacta (SNpc) of sagittal mouse brain sections 5 weeks after AAV-tetO-Venus virus injection. TH-tTA mice were treated with DOX for 10 days before streotactic injections until analysis to switch the system off (A). TH-rtTA mice were here not DOX treated to keep the system inactive (B). Non-transgenic control mice were analyzed also 5 weeks after AAV-tetO-Venus virus injection (C). Scale bars: 250 μm. (TIF) [file pone.0136203.s007.tif]
